# Prevalence and associated factors of ever use of electronic cigarettes: Findings from a hospitals and health clinics study based in Malaysia

**DOI:** 10.18332/tid/99258

**Published:** 2018-11-23

**Authors:** Komathi Perialathan, Abu B. Rahman, Kuang H. Lim, Yusoff Adon, Azman Ahmad, Nurashma Juatan, Norrafizah Jaafar

**Affiliations:** 1 Institute for Health Behavioural Research, Kuala Lumpur, Malaysia; 2 Institute for Medical Research, Kuala Lumpur, Malaysia; 3 Raja Permaisuri Bainun Hospital, Ipoh, Malaysia

**Keywords:** electronic cigarette, perceptions, hospital-based study, smoking status

## Abstract

**INTRODUCTION:**

Electronic cigarettes (e-cigarettes) are new smoking devices that have gained popularity recently. However, there is limited evidence on e-cigarette consumption in Malaysia. This study aims to determine the prevalence, risk factors and perception associated with e-cigarette use among those attending government hospitals and health clinics in Malaysia.

**METHODS:**

A cross-sectional hospital-based study was conducted in seven public hospitals and health clinics in Malaysia, which were selected through a two-stage cluster sampling. A validated questionnaire was used to obtain data from the selected participants. Multivariable logistic regression was employed to determine the association between sociodemographics and perceptions of e-cigarette use.

**RESULTS:**

Almost three-quarters (73.6%; n=923/1254) of participants were aware of e-cigarettes and 13.2% (n=122/923) reported having ever used e-cigarettes. The prevalence was significantly higher among males (18.1%), smokers (21.4%), and younger age group 18–34 years (30.2%). Ever users showed favourable perceptions towards e-cigarettes compared to non-users (23.3% vs 30.14%, p<0.001). Multivariable logistic regression revealed that current smokers, younger age group and those possessing a positive perception towards e-cigarettes were likely to be ever users of e-cigarettes.

**CONCLUSIONS:**

The study showed that the awareness level of e-cigarettes was high amongst the population but the prevalence of ever e-cigarette user was moderate. Most of the ever e-cigarette users were male, current smokers, young adults and those with favourable perceptions towards e-cigarettes. Therefore, effective health educational activities regarding safe usage of e-cigarettes targeting those group identified in this study are warranted to reduce the negative outcomes from the use of this product.

## INTRODUCTION

Electronic cigarettes (e-cigarettes), also referred to as electronic nicotine delivery system (ENDS), are hand-held battery-operated devices that provide inhaled doses of vaporized nicotine^1^. These devices were introduced by the Chinese company Ruyan in 2004 and gained large popularity and rapid increase around the world in recent years^2^. Surveys in 7 countries (Russia, Germany, United Kingdom, South Korea, Italy, Poland and France) revealed that the numbers of e-cigarette users increased from 2.8 million in 2013 to 5.1 million in 2015^3^. In 2013, the global market for e-cigarette revenue was around $2 billion and is expected to rise to $10 billion by 2017^4^.

Proponents and manufacturers claimed that e-cigarettes are healthier alternatives to conventional cigarettes and can be used as smoking cessation devices^5^. Such claims influenced, to a certain extent, customers, especially smokers, to use them as a quit-smoking aid, as stated by Ramo et al.^6^. Christensen et al.^7^ found that the most common reasons for smokers initiating e-cigarette use were to reduce the harm of conventional cigarettes or to help quit cigarette smoking^8^. However, some studies debated the ability of e-cigarettes to be quit-smoking aids. Adkison et al.^9^ revealed no difference in cessation between e-cigarette users and non-users. In addition, Vickerman et al.^10^ showed that e-cigarette user groups (used e-cigarettes for one month or more, used e-cigarettes for less than one month) were significantly less likely to abstain from tobacco compared with participants who had never tried e-cigarettes (21.7% and 16.6% vs 31.3%, respectively).

Many studies have revealed ever use of e-cigarettes among adolescents as one of the determinants for their subsequent cigarette smoking habit. A systematic review and meta-analysis on longitudinal studies comprising 17389 adolescents and young adults by Soneji et al.^11^ showed that e-cigarette use increases the risk of smoking initiation among adolescents, whereby the adjusted pooled odds ratio for subsequent cigarette smoking initiation was 3.50 (95% CI: 2.38–5.16) for ever versus never e-cigarette users. Another longitudinal study by East et al.^12^ revealed that there will be higher likelihood of smoking initiation among adolescents who are ever e-cigarette users (adjusted odds ratio, AOR=1.89, 95% CI: 3.56–39.72). This is worrisome in view of almost one-fifth (19.1%, 95% CI: 17.4–21.0) of Malaysian school going adolescents of age 10–19 years are ever e-cigarette/vape users whereby 46.5% (95% CI: 39.8–53.4) of ever e-cigarette/vape users tried their first e-cigarette/vape before the age of 14 years. The same study stated the prevalence of current dual users of e-cigarettes/vape and cigarettes as 5.4%, out of which 10.8% started with e-cigarettes/vape before cigarettes, which shows that the initiation to smoking started from e-cigarette use^13^. The negative outcome of smoking initiation among non-smoking adolescents or young adults is that they eventually will become smokers and at risk to the known health burdens of combustible tobacco cigarettes^14,15^.

Various studies have been carried out to determine the factors associated with e-cigarette use. Choi and Forster^16^ revealed that male, younger age, smokers, and participants who had at least one close friend who smoked, were more likely to have used e-cigarettes. Giovenco et al.^17^ reported that the younger age group, white and non-Hispanic, were more likely to ever use e-cigarettes. In their study in 27 European Union member states, Filippidis et al.^18^ found that the younger age group living in urban areas and higher education were associated with a higher likelihood of being an e-cigarette user. Adkison et al.^9^ showed that the prevalence of trying e-cigarettes was higher among younger, non-daily smokers with a high income and among those who perceived e-cigarettes as less harmful than traditional cigarettes. Current usage was higher among both non-daily and heavy (≥20 cigarettes per day) smokers. In all, 79.8% reported using e-cigarettes because they were considered less harmful than traditional cigarettes; 75.4% stated that they used e-cigarettes to help them reduce their smoking; and 85.1% reported using e-cigarettes to help them quit smoking.

In Malaysia, Global Adult Tobacco Surveys (GATS) - Malaysia 2011^19^ revealed that 20.5% were aware of e-cigarettes but only a small proportion (3.2%) used them. However, in 2015, the National Health and Morbidity Survey (NHMS) 2015^20^ reported 10.9% in the use of smokeless tobacco products, including e-cigarettes among Malaysian adults. The increasing trend is worrisome, in view of smokeless tobacco use including e-cigarettes, as it might encourage smoking initiation among non-smokers, long-term dual usage among current smokers, the resumption of smoking among former smokers and the maintenance of nicotine addiction (GATS)^19^. Thus, there is a need to carry out a study to address these issues. This study aimed to determine the prevalence of e-cigarette use and the association of sociodemographics and perceptions with e-cigarette use.

## METHODS

### Study design

A cross-sectional study design and two stage cluster sampling was used to select the participant. The study was carried out from June 2014 to July 2015. First, the public hospital and clinics were stratified according to six zones: southern (Johor and Malacca), central (Selangor, Wilayah Persekutuan Kuala Lumpur, Wilayah Persekutuan Putrajaya and Negeri Sembilan), northern (Perak, Penang, Kedah and Perlis), east coast (Pahang, Kelantan and Terengganu), Sabah and Sarawak. Second, there was a selection of one or two public hospitals and health clinics from each zone via a simple random sampling method. The sample size was determined based on prevalence of e-cigarette use from GATS-M 2011^19^, which is 3.2%. A 2.5% precision/accuracy rate was used (precision/accuracy was defined as — how close observed sample statistic comes to the true population parameter). Design effect of 3.137 was used to cater for clustering effect from each locality. The sample obtained was multiplied by two in view of two stages of stratification in the sampling, and an expected non-response rate of 10%. Based on these parameters, a total of 1318 participants were required for the study.

The intercept approach was used in this study. Trained research team members approached every third person passing by the interview station to participate; if the respondent did not fulfil the inclusion criteria or refused to participate, the next person passing by was selected. The self-administered questionnaire was distributed to the participants after they consented in writing. The research team members assisted the participants who faced difficulties in understanding certain items in the questionnaire. Ethical approval was granted by the Medical Research Ethical Committee, Ministry of Health, Malaysia (NMRR–14–1117–19586).

### Study measurement

A questionnaire was developed by research team members comprising academicians and researchers from local universities and research institutions in Malaysia. The questionnaire was pre-tested in Bangsar, Kuala Lumpur, and minor corrections were made based on the feedback from the pretest. The questionnaire contains questions on sociodemographic background, awareness of e-cigarettes, status of e-cigarettes, cigarette smoking status and perception of e-cigarettes.

Awareness of e-cigarettes was measured using the question: ‘Do you know about e-cigarette’; with the response choices: 1) ‘I know about it but did not used it’, 2) ‘I know EC and used it as smoking cessation tool’, 3) ‘I know about it and used together with cigarette’, and 4) ‘Never heard of it’. Participants who selected choice 4) were excluded from further analysis. Dependent variables of e-cigarette use were measured using the question: ‘Do you currently use e-cigarettes?’; with the response choices: ‘Daily’, ‘Occasionally’, ‘Ever used but quit already’ and ‘Not at all’. Participants who answered ‘Not at all’ were classified as ‘never e-cigarette user’; those who answered ‘Daily’, ‘Occasional’ and ‘Ever used’ were classified as ‘ever e-cigarette user’.

Independent variable was smoking status of participants (current smoker: smoking daily or occasionally and non-smoker: never smoke a cigarette). Perceptions towards e-cigarettes were measured using 14 items (see Appendix) (e.g. smoker should be encouraged to use e-cigarette to quit smoking) and a Likert-type scale (‘strongly disagree’, ‘disagree’, ‘agree’ and ‘strongly agree’). All the negative items were recoded before the score. A higher score indicated a positive perception towards e-cigarettes. Sociodemographics, such as gender (male, female), ethnicity (Malay, Chinese, Indian, other indigenous groups, others), educational attainment (no formal education, primary, secondary, tertiary education), age group (18–24, 25–34, 35–44, 45 years and older) and marital status (single, married, widow/widower/separated) were also included in the analysis.

### Data analysis

Data were cleaned prior to analysis. Descriptive statistics were used to describe the sociodemographic characteristics of participants, smoking status and their perceptions towards e-cigarettes. Chi-squared analysis was used to test the association between the use of e-cigarettes, sociodemographic variables and smoking status. An independent sample t-test was employed to test the mean difference of perceptions score towards e-cigarettes by e-cigarette user. All bivariate analyses with p≤0.25 were included in the multivariable logistic regression. The final model was tested using Hosmer–Lemeshow analysis; the p-value of 0.682 indicated the model is a good fit. All possible two-way interactions between the independent variables in the final model were also analysed and analysis showed that p>0.05 indicated no significant two-way interaction between independent variables in final model. All statistical analyses were run at 95% confidence interval (CI) using SPSS statistical software version 22.0.

## RESULTS

A total of 1254 participants responded to the study. Given the response rate of 95.1%, the proportion of male participants was more than two times higher than that of females (69.7% vs 30.1%). Two-thirds of the participants were Malays (65.9%), followed by other races (Bumiputra Sabah, Sarawak and others, 22.4%), Chinese (6.7%) and Indian (5.0%). Out of the participants, 66.2% were single, widow or widower, and one-third had higher than a secondary school education. Almost half of the participants were current smokers. However, 331 respondents were excluded as they were unable to proceed further with the questionnaire to answer detail items on e-cigarette awareness and use. Out of 923 participants, 13.2% had ever used e-cigarettes. Chi-squared statistical tests showed a significant higher usage of e-cigarettes among males compared to females (18.1% vs 0.4%, p<0.001). In addition, bivariate analysis revealed a significant association between ethnic groups, age groups, smoking status and ever e-cigarette users. However, no similar association was observed between education level, marital status, and ever e-cigarette user. There was a significant difference in the score of perceptions toward e-cigarettes among non-users (M=23.30, SD=10.33) and ever users (M=30.14, SD=12.69) (t=5.57, p<0.001), which indicates those who have used e-cigarettes tend to accept or be positive about the use of e-cigarettes (Table 1).

**Table 1:** Sociodemographic characteristics of participants, and status of ever e-cigarette (EC) user by sociodemographics and smoking status

*Variable*	*Participants*		*Status of ever EC user*				*χ^2^*	*p*
			*Yes*		*No*			

	*n*	*%*	*n*	*%*	*n*	*%*		
**Overall**	1254	100.0	122	13.2	801	86.8		
**Gender**								
Male	874	69.7	121	18.1	546	81.9	50.81	<0.001
Female	380	30.3	1	0.4	255	99.6		
**Ethnic**								
Malay	826	65.9	95	15.0	539	85.0	8.16	0.04
Chinese	64	6.7	2	3.8	51	96.2		
Indian	63	5.0	2	5.7	33	94.3		
Others	291	22.4	23	11.4	178	88.6		
**Age Group (years)**								
18–24	224	17.3	26	15.2	145	84.8	10.37	0.016
25–34	422	33.7	51	13.0	289	85.0		
35–44	317	25.3	34	14.8	195	85.2		
45 and older	291	23.2	11	6.0	172	94.0		
**Education attainment**								
Until secondary school	737	65.7	59	11.6	448	88.4	3.38	0.066
College and tertiary	384	34.3	49	16.2	250	83.8		
**Marital status**								
Married	422	33.8	77	12.6	536	87.4	0.75	0.387
Single/widow(er)	828	66.2	45	14.6	263	85.4		
**Smoking status**								
Current smoker	564	45.8	102	21.9	363	78.1	62.09	<0.001
Non-smoker	690	55.0	20	4.4	436	95.6		

Note: Only 923 participants who aware of EC were included in the analysis of ever EC user.

Female participants were excluded from the multivariable analysis in view of the single person who was ever e-cigarette user (n=1). The multivariable logistic regression among males showed that the odds of the younger male age groups using e-cigarettes was 4 times that of the oldest age group (AOR=4.02, 95% CI: 1.50–10.80). The odds of current smoking males using e-cigarettes was 3.5 times that of non-smoking males (AOR=3.46, 95% CI: 1.85–6.50). Every point/unit increase in the positive perception score increased the likelihood of e-cigarette use by 6%. In addition, participants with higher education attainment were more likely to become ever e-cigarette users (AOR=1.69, 95% CI: 1.05–2.72), shown in Table 2.

**Table 2:** Multivariable logistic regression analysis to determine the association between sociodemographics and smoking status with male ever EC user

*Variable*	*Adjusted Odds Ratio*	*95% CI*	
**Ethnic**			
Malay	1		
Chinese	0.22	0.03	1.80
Indian	0.69	0.14	3.41
Others	0.80	0.45	1.40
**Age Group (years)**			
18–24	4.02	1.50	10.80
25–34	3.22	1.43	7.26
35–44	2.92	1.28	6.65
45 and older	1		
**Education attainment**			
No formal education - secondary school	1		
College and tertiary	1.69	1.05	2.72
**Smoking status**			
Current smoker	3.46	1.85	6.50
Non-smoker	1		
**Marital status**			
Married	1		
Single	1.06	0.60	1.86
Perception score toward e-cigarette	1.06	1.03	1.08

Hosmer–Lemeshow test, chi-squared value 5.67, df (8), p=0.682. Note: The multivariate analysis is for male participants, as female participants were dropped as there was only 1 female ever e-cigarette (EC) user.

## DISCUSSION

To our knowledge this is a first study in Malaysia to describe e-cigarette use among Malaysian adults in hospital/health care facilities in Malaysia. The study showed that the overall prevalence of ever e-cigarette users was 13.2%, which was similar to the prevalence of 11.9% reported in the recent National E-Cigarette Survey, Malaysia^21^. Among adults in the United States, the prevalence is 12.6%^22^ and in 27 European countries the prevalence is 11.6%, as reported by Filippidis et al.^18^. The prevalence was relatively lower than in an International Tobacco Control study of 10 countries conducted by Gravely et al.^3^ that reported a prevalence of 19%. In contrast, the prevalence among adults in this study was almost five-fold higher than the prevalence of 2.7% and 2.3% reported by Chang et al.^23^ in Taiwan and Jiang et al.^24^ in Hong Kong, respectively. The differences in the prevalence of ever e-cigarette users between the countries might be due to the level of regulations and enforcement of e-cigarettes.

The prevalence of e-cigarette users was significantly higher among males compared to females (18.4% vs 0.4%), which was similar to the trend of tobacco product usage^19^. This finding was consistent with a study of adults by Elkalmi et al.^25^ in Kuantan, Malaysia, which reported 91.7% of e-cigarette use among males and only 8.3% among females. In addition, in 2017 Chang et al.^23^ also reported that the prevalence of e-cigarette use among males was almost five-fold higher than for females in Taiwan (4.6% vs 0.9%), and a similar trend of e-cigarette and other tobacco product usage was also observed for gender in Canada^26^.

We envisage in Malaysia that the finding might be due to similar reasons quoted by Tsai et al.^27^, which stated that Asian social norms do not favour smoking among females and e-cigarette use is considered a type of smoking. In view of Malaysians being a collective community, whereby the norm of society precedes the choice of individual, the individual will embrace and behave in accordance with the community norm. Our finding also showed higher ever use of e-cigarettes among younger age groups, which is consistent with the findings by Chang et al.^23^ and Li et al.^28^ among adults in Taiwan, United States and New Zealand, respectively. Similar findings were also reported in Canada, in which the prevalence was 20.1% among adults of age 20–24 years compared with 3.7% among those 45 years and older^26^.

The findings in our study is expected because of the greater receptiveness of the younger age group of participants to new innovations or products compared to the older age group, as reported by Helm and Landschulzet^29^ in their study on consumer age and desire for products and brands. Another plausible reason for our findings can be associated with marketing strategies by e-cigarette companies, which target youth through Internet and social media. Extensive future studies are recommended to investigate this aspect, as proper regulations are needed to the extent that the marketing of products can influence young people and later lead them to affect their own health.

Multivariable logistic regression analysis revealed that participants with higher education attainment were more likely to be ever e-cigarette users compared with those with lower education levels (AOR=1.69, 95% CI: 1.05–2.72). This finding is in-line with the study on US adults by Pearson et al.^30^ who found that the likelihood of e-cigarette use was higher among participants with college degree attainment compared to those with less than a high school diploma (AOR=2.72, 95% CI: 1.33–5.59). Similarly, Wang et al.^31^ also reported that participants with tertiary education level are 2.78 times (95% CI: 1.17–6.60) more likely to be ever e-cigarette users compared with their counterparts of lower education level.

Both studies^30,31^ revealed a positive relationship between level of education and use of e-cigarettes. Participants with higher education attainment might have had early access to the latest phenomena and lifestyle trends; they could afford to be engaged with a costly habit and this phenomenon was indicated with Lopez et al.’s smoking epidemic model^32^. This model stated that smoking emerges first among high status groups, who are more open to innovations and have the resources to adopt them first. A large literature on diffusion of innovations recognizes the tendency for high status persons to most quickly adopt new ideas and behaviours^33^. In a study analysing diffusion, cohort change and social patterns of smoking, Pampel^34^ stated that the diffusion of the use of manufactured cigarettes, being both technological and cultural innovations, follows such a status-based pattern. High status groups begin smoking earlier than the general public.

Smoking status was a significant associated factor with ever use of e-cigarettes (AOR=3.46, 95% CI: 1.85–6.50). Choi and Forster^16^ showed that a current smoker has a greater propensity to use e-cigarettes compared to a non-smoker. Other studies have suggested that smokers turn to e-cigarettes in an attempt to quit smoking, reduce number of cigarettes smoked and save the expense of buying conventional cigarettes, which might explain the findings in our study^35^. This trend is clearly explained through diffusion of innovation theory by Rogers^36^, which posits that an individual with prolonged existing behaviour is more receptive to adopt and try the new behaviour, which has some similar characteristic, idea or product that spreads through a population or social system.

The study showed significant association between positive perception towards e-cigarettes and ever use of e-cigarettes (AOR=1.06 95% CI: 1.03–1.08). Similar findings are also reported by Choi and Forster^16^ who stated that ever e-cigarette users tend to perceive e-cigarettes as less harmful, less addictive and facilitate in the smoking cessation process. This finding is in line with the Health Belief Model by Hochbaum, which posits that an individual who perceives the benefit of certain behaviour will more likely accept and practice it^37^. Lim et al.^38^ supported this finding, reporting that knowledge and perception influences the actual behaviour of smoking among Malaysian adults.

### Limitations

There were several limitations in this study. Firstly, the hospital-based sample might not be representative of the general population of Malaysia. Secondly, the status of e-cigarettes was self-reported, which might be under or over-reported due to recall bias. Thirdly, the causal relationship cannot be established due to the cross-sectional study design. Fourthly, 331 (26%) participants had to be excluded because they were not aware of e-cigarettes, and we did not rule out the possibility that this might affect the result, especially on the distribution of gender, age group and educational status between those who participated and those who did not, as the sampling was not totally random. Lastly, variables such as having children in the house, shown to be associated with e-cigarette use was not investigated in the current study^39^.

## CONCLUSIONS

Overall, the findings of this study highlight that a substantial proportion of participants who are male, being a current smoker, from younger age group, having higher education attainment and positive perceptions towards e-cigarettes use them. In addition, the study also found that the majority of participants in our study were aware of e-cigarettes, although a product new to the market. As there is no scientific evidence that e-cigarette use is safer than cigarette smoking^40^, it is important to disseminate relevant information regarding e-cigarettes and the possible harms, especially to the population with a propensity to ever use e-cigarettes, as identified in this study. In summary, additional research is needed to understand the patterns of e-cigarette usage amongst a representative sample of the Malaysian population, especially in terms of prevalence, behaviours associated with its use, dual usage of tobacco product and e-cigarettes, it’s effectiveness as a cessation aid, and main factors for its initiation, especially among non-smokers.
