# Supplementary material for: Prevalence and associated factors of ever use of electronic cigarettes: Findings from a hospitals and health clinics study based in Malaysia
Source: Tob Induc Dis. 2018 Nov 23;16:55. doi: 10.18332/tid/99258 (PMC6659488; doi:10.18332/tid/99258)
Supplement: Supplementary file 1 [file TID-16-55-s1.pdf]

Appendix. Perception towards EC

Items	Strongly disagree *Sangat setuju*	Disagree *Tidak setuju*	Agree *Setuju*	Strongly agree *Sangat setuju*
Family members should encourage smokers to switch to E-cig instead as it is safer. *Ahli keluarga perlu menggalakkan perokok bertukar kepada E-cig kerana ia lebih selamat*				
Smokers are encouraged to use E-cig to aid in quitting smoking. *Perokok digalakkan menggunakan e-cig untuk membantu berhenti merokok*				
E- Cigarette is the main choice for effective quit smoking method. *E-cig adalah pilihan utama kaedah berhenti merokok yang berkesan*				
E-cig is more effective than normal medication therapy to quit smoking. *E-cig lebih berkesan dari terapi ubat berhenti merokok*				
E-cig should be allowed to be used at non-smoking area. *E-cig boleh dibenarkan di kawasan larangan merokok*				
E-cig should be banned in Malaysia *E-cig perlu diharamkan di Malaysia*				
E-cig doesn't cause any harmful effects to chronic disease patients who are diagnosed with diabetes, hypertension and cardiac problem. *E-cig tidak memberi kesan mudarat kepada pesakit kronik (diabetes, jantung, darah tinggi).*				
E-cig is not harmful to users. *E-cig tidak membahayakan kesihatan penghisap*				
Smokes from e-cig are not harmful to others. *Asap e-cig tidak membahayakan orang lain.*				

Sharing of e-cig can cause transmission of infectious diseases (example Tuberculosis (TB), influenza)<br>*Berkongsi e-cig dengan pengguna orang lain boleh menyebabkan jangkitan penyakit (contoh batuk kering, influenza)*				
E-cig gives the same satisfaction as conventional cigarette.<br>*E-cig memberi kepuasan yang sama dengan rokok biasa*				
E-cig became the choice as the cost is cheaper.<br>*E-cig menjadi pilihan kerana kosnya menjimatkan*				
E-cig is more accepted by people around compared to conventional cigarette.<br>*E-cig boleh diterima oleh orang sekeliling berbanding rokok biasa*				
Availability of various flavours in e-liquid will encourage even non-smokers to try it.<br>*Perisa (flavor) yang pelbagai dalam e-liquid menggalakkan bukan perokok untuk mencuba*				

© 2018 Perialathan K.
